# *Taenia solium* Cysticercosis: The Case of Cuba

**DOI:** 10.1371/journal.pntd.0002202

**Published:** 2013-07-11

**Authors:** Kirezi Kanobana, Aniran Ruiz, Lazara Rojas, Rene Andrade, Felix Rosado, Katja Polman, Fidel Angel Núñez

**Affiliations:** 1 Institute of Tropical Medicine, Antwerp, Belgium; 2 Institute of Tropical Medicine “Pedro Kourí”, Havana, Cuba; 3 Institute of Neurology and Neurosurgery, Havana, Cuba; Universidad Peruana Cayetano Heredia, Perú

Cysticercosis is a neglected zoonotic disease attributable to infection with the metacestode larval stage of the tapeworm *Taenia solium*. Humans are the sole final hosts, carrying the adult tapeworm in the intestine (taeniosis). Pigs are the main intermediate host, but humans also may accidentally get infected through the ingestion of *T. solium* eggs (shed in human feces) that later develop into oncospheres. When cyst formation occurs in the brain, the disease is called neurocysticercosis (NCC), which is increasingly recognized as the leading cause of epilepsy in large parts of Africa, Asia, and Latin America [1]. Cysticercosis is a poverty-associated disease, and its burden is known to be substantial in the Latin American continent [2]–[4]. On the other hand, based on limited numbers and often outdated reports, cysticercosis does not seem to be a common disease in the Caribbean [5], with the exception of Haiti, where several case reports have been published in the past decades [6],[7]. Also, a recently conducted cross-sectional serological survey in Port-au-Prince revealed prevalences of cysticercal antibodies of 2.8% (6/216) in healthy adults [8], which is comparable to those found in some Latin American and African countries. Here, we compiled the available information on cysticercosis/taeniosis in Cuba and supplemented this with data from a recently conducted survey on NCC in patients with epilepsy in order to evaluate the importance of *Taenia solium* cysticercosis in the country.

A literature search was conducted in the MEDLINE, PUBMED, LILACS (Latin American and Caribbean Health Science database, http://lilacs.bvsalud.org/en/), and CUMED databases with the keywords *Taenia solium*, cysticercosis, Cuba, and neurocysticercosis on October 15, 2012. In addition, references of retrieved reports were checked for additional information. The outcome is summarized in Table 1. Only a few case reports exist, and two dedicated surveys, spread over a period of more than 70 years. A case report from 1984 describes the identification of a *T. solium* tapeworm upon treatment of a Cuban man who had recently travelled to Angola [9]. The first case report on human cysticercosis dates from 1901 and is considered the first report ever on the disease for Mexico and Cuba. The author describes the history of a Cuban patient who died in a psychiatric asylum in Mexico and multiple cysts were found during autopsy [10]. Since then, only four additional case reports have been published: two in 1967 [11] and three more than twenty years thereafter, one in 1989 [12] and two in 1991 [13]. In most cases, cysticercosis was confirmed by histopathology (Table 1).

**Table 1:** Chronological overview of reports on taeniosis/cysticercosis in Cuba.

Date	Publication Type	Location	Summary	Reference
1901	Case report (n = 1)	Mexico	A female patient from Cuba who died in a psychiatric asylum in Mexico. During autopsy, multiple cysts were found. The country where the parasitic infection was acquired is not clarified.	[10]
1967	Cases report (n = 2)	Camagüey, Baracoa	A 23-year-old woman from Camagüey with antecedents of convulsions without medical treatment for 15 years, and a 39-year-old man from Baracoa complaining of headache and vomiting for more than a year. The man was hospitalized because of signs of increasing intracranial pressure and visual disturbances. Both cases were associated with a history of eating raw pork and the diagnosis was confirmed by the detection of cysts in brain biopsies.	[11]
1984	Case report (n = 1)	History of travel to Angola	A 26-year-old male, coming back from Angola, presenting with abdominal pain, loss of appetite, and drowsiness, with a history of eating pork on regular basis.	[9]
1989	Case report (n = 1)	Camagüey	*	[12]
1991	Cases report (n = 2)	Baracoa	A 29-year-old woman with antecedents of headache, vomiting, and weight loss during the nine months prior to hospital admission. She died in the neurology ward. Cysticercosis was confirmed by postmortem histopathology, which revealed the presence of hooks n the invaginated scolex of one brain cyst.A 10-year-old girl with a palpable subcutaneous cyst on her back. Cysticercosis was confirmed by histopathology upon surgical removal of the nodule.	[13]
1999	Retrospective study (n = 5/33,424)	Cuba	Retrospective review of clinical cases of patients admitted to the INNH at Havana in the period from 1964 to 1989 was conducted in search of patients diagnosed with NCC. Five patients, of whom three with a history of travel and two of non-Cuban origin, were identified. The clinical history of the cases is described.	[14]
2001	Retrospective serological survey (n = 0/384)	Havana	Detection of antibodies in cerebrospinal fluid of children attending the emergency ward of the Hospital Pediátrico San Miguel del Padrón in Havana because of seizures. The cohort included children with and without fever with a mean age of 5.6 years. Cysticercal antibodies were detected by a complement fixation assay.	[16]
2010	Serological survey (n = 158)	Havana	Cohort of 158 PWE (age range: 5–76, 41% males and 59% females) attending the epilepsy consult of the INNH; 21 were suspected of NCC based on epidemiological, clinical, or neuroimaging (CT-scan) grounds. There were no demographic differences between the PWE suspected of NCC and the PWE not suspected of NCC. Serology outcomes by EITB and antigen-ELISA were negative in all patients.	**

This table summarizes the outcome of a literature search in the MEDLINE, PUBMED, LILACS (Latin American and Caribbean Health Science database, http://lilacs.bvsalud.org/en/), and CUMED databases conducted with the keywords *Taenia solium*, cysticercosis, Cuba, and neurocysticercosis on October 15, 2012. In addition, references of retrieved reports were checked for additional information.

* The title of the publication points to a case report of cerebral cysticercosis, but details on the case could not be obtained.

** This study was recently conducted by our group (unpublished). PWE = people with epilepsy. NCC = neurocysticercosis.

In 1999, a retrospective study reviewed clinical cases of patients admitted to the Instituto de Neurología y Neurocirugía in Havana (INNH, which is the reference centre for epilepsy in Cuba) between 1964 and 1989, in search of patients diagnosed with NCC [14]. Only five patients with confirmed NCC were identified out of the 33,424 admissions over 25 years. Three of the patients were foreigners, and there was evidence that the two Cuban patients had acquired the disease abroad. The authors concluded that cysticercosis was absent in Cuba. This was followed by a reply a few years later [15], urging not to rush to such firm conclusions. The authors stated that NCC was not absent in Cuba, but very rare, and referred to their earlier case reports [11]–[13]. They also mentioned the habit of pork consumption in the country, and emphasized the need for continuous education to prevent disease transmission. The last published study on cysticercosis in Cuba is a serological survey conducted in 2001. The presence of *T. solium* cysticercal antibodies was assessed in the cerebrospinal fluid of 384 children who had attended the emergency ward of a hospital because of seizures. None of them was found positive [16]. However, the origin of the seizures and the epidemiological antecedents of the children were unknown, and little is known about the sensitivity and specificity of the diagnostic assay used.

National surveys on intestinal helminth infections detected no or only few *Taenia* spp. eggs (reviewed by [17]). The most recent survey conducted in 2009 detected only six cases infected with *Taenia* spp out of the 5,850 individuals surveyed [18]. There are no reports on porcine cysticercosis in Cuba, and it has thus far never been detected in abattoir screenings (Dr. Mendez and Dr. Rafmari, Instituto de Medicina Veterinaria, personal communication).

Recently, the INNH requested our assistance for the diagnosis of NCC in a group of patients with unknown causes of epilepsy. To this end, we combined two serological tests: 1) the detection of antibodies by enzyme immuno transfer blot (EITB), which is the gold standard for serodiagnosis of cysticercosis but does not allow differentiation between past and current infection; and 2) the detection of circulating cysticercal antigen by ELISA, which has a high sensitivity for the detection of viable and colloidal cysts [19].

The cohort consisted of 158 people with epilepsy (PWE), above five years of age, who attended the epilepsy consult in the period from January to July 2010 and were followed up by the INNH. Twenty one of these patients were suspected of NCC based on clinical, epidemiological, or neuroimaging grounds [20]. Epilepsy was defined as two or more nonfebrile unprovoked seizures, occurring at least 24 hours apart. Computer tomography (CT) was not conducted at the time of the study, but CT scans were available from previous investigations. Ethical clearance for the study was obtained by the Internal Review Board of the Institute of Tropical Medicine in Antwerp (ref. 09395682) and the ethical committees of the INNH, the Instituto Pedro Kouri, and the University Antwerp, Belgium (approbation number 9/47/262).

There were no major differences in gender, age, ethnicity, and residence between the PWE suspected of NCC and the PWE who were not suspected. Irrespective of whether they were suspected of NCC, in the majority of the PWE, the onset of seizures had started more than one year prior (approximately 30%, 20%, and 40% of the PWE for 1–10 years, 11–20 years, and 21–30 years, respectively). Serology outcomes by EITB and antigen-ELISA were negative in all patients, suggesting a lack of association of NCC with epilepsy in these patients (see Table 1).

Taken together, the above data suggest that taeniosis/cysticercosis is not endemic in Cuba. The case reports are few, but illustrate that autochthonous NCC may occur, albeit focally. Baracoa, where three of the five autochthonous cases of cysticercosis were diagnosed, is remotely located on the eastern end of the island, close to Haiti. Until the Cuban revolution, it was entirely isolated and could only be reached by sea. Nowadays, it is frequented by Haitian immigrants who accidently arrive at the Cuban coasts while trying to reach U.S. territory. Hence, it is very much possible that these cases were acquired by human to human transmission of *T. solium* eggs from Haitian immigrants.

Despite the few reports on cysticercosis and taeniosis, health policy makers in Cuba remain cautious, which was illustrated by the establishment of a National Programme dedicated to the control of *Taenia* spp. (Programa Nacional para el Control de Céstodes, Repùblica de Cuba, referred to by [15]). The occurrence of sporadic cases in Cuban regions bordering Haiti, the increasing migration from Latin American countries where the disease is endemic, and the expanding tourism in Cuba [21] pose a potential threat of importing the disease. To keep a cysticercosis-free status, continued vigilance among veterinary and public health officers is warranted.
